# Microbial Metal Resistance within Structured Environments Is Inversely Related to Environmental Pore Size

**DOI:** 10.1128/AEM.01005-21

**Published:** 2021-09-28

**Authors:** Harry J. Harvey, Anna M. T. Mitzakoff, Ricky D. Wildman, Sacha J. Mooney, Simon V. Avery

**Affiliations:** a School of Life Sciences, University of Nottingham, Nottingham, United Kingdom; b Faculty of Engineering, University of Nottingham, Nottingham, United Kingdom; c School of Biosciences, University of Nottingham, Nottingham, United Kingdom; Nanjing Agricultural University

**Keywords:** environmental structure, stress resistance, yeast, additive manufacturing, pore space, lead toxicity, microbial stress response, soil

## Abstract

The physical environments in which microorganisms naturally reside rarely have homogeneous structure, and changes in their porous architecture may have effects on microbial activities that are not typically captured in conventional laboratory studies. In this study, to investigate the influence of environmental structure on microbial responses to stress, we constructed structured environments with different pore properties (determined by X-ray computed tomography). First, using glass beads in different arrangements and inoculated with the soil yeast Saitozyma podzolica, increases in the average equivalent spherical diameters (ESD) of a structure’s porous architecture led to decreased survival of the yeast under a toxic metal challenge with lead nitrate. This relationship was reproduced when yeasts were introduced into additively manufactured lattice structures, comprising regular arrays with ESDs comparable to those of the bead structures. The pore ESD dependency of metal resistance was not attributable to differences in cell density in microenvironments delimited by different pore sizes, supporting the inference that pore size specifically was the important parameter in determining survival of stress. These findings highlight the importance of the physical architecture of an organism’s immediate environment for its response to environmental perturbation, while offering new tools for investigating these interactions in the laboratory.

**IMPORTANCE** Interactions between cells and their structured environments are poorly understood but have significant implications for organismal success in both natural and nonnatural settings. This work used a multidisciplinary approach to develop laboratory models with which the influence of a key parameter of environmental structure—pore size—on cell activities can be dissected. Using these new methods in tandem with additive manufacturing, we demonstrated that resistance of yeast soil isolates to stress (from a common metal pollutant) is inversely related to pore size of their environment. This has important ramifications for understanding how microorganisms respond to stress in different environments. The findings also establish new pathways for resolving the effects of physical environment on microbial activity, enabling important understanding that is not readily attainable with traditional bulk sampling and analysis approaches.

## INTRODUCTION

The physical environments in which microorganisms reside are usually complex and dynamic, from the micrometer scale (e.g., distances between individual cells during biofilm formation [1]) to the meter scale (e.g., governing community dynamics in soils [2–4]). Structural heterogeneity within an environment (such as the uneven separation of habitable space by physical barriers) is often coupled with heterogeneity in the spatial distribution and concentration of other environmental components, such as nutrients, oxygen, and secreted metabolites like cell-cell signaling molecules (5–7). Gradients in these parameters can facilitate the division of labor or resilience to perturbation within microbial populations, often creating site-specific niches which can propagate microbial diversity within the broader system (8, 9). Such interactions between microorganisms and structured environments can have important ramifications for their success in both natural and nonnatural settings, such as microscale surface topologies that facilitate biofilm formation and persistence (10, 11) or soil particles and aggregates giving protection to soil organisms from pollutants (8, 12, 13).

The soil matrix provides one well-studied but incompletely understood example of a structured environment, considered to be “the most complicated biomaterial on the planet” (14). This is in part due to variation in the diameters, shapes, and connectivity of soil pores (15). Soils offer particularly varied types of structure and ranges of pore space, which can impinge on the biogeochemical cycling activities that they support (5). Whereas there is evidence for differential abundances of nutrients or pollutants within different soil fractions (16, 17), measurements of these parameters in soil samples are often considered representative of the whole sample. Consequently, the impact of structured environments on the distribution of organisms and organic or inorganic compounds at the micro- or millimeter scale of microbial niches requires further research attention. Furthermore, how the structure of an organism’s immediate environment, such as the surrounding soil architecture, impacts its response to abiotic factors (e.g., stressors) is poorly characterized.

Previous studies have attempted to understand the impact of environmental structure on microbial cell-cell interactions (18–20). However, few studies have dissected the impact of physical porous structure on microbial stress resistance in a controlled laboratory setup. Because pore size can affect the rate of movement and diffusion of fluids through a porous medium (often termed hydraulic conductivity) (21), we hypothesized that environmental structure, and pore size specifically, would influence the exposure of microorganisms to an imposed stressor within the liquid phase. In this study, to test this hypothesis, we built glass bead structures and additively manufactured lattice structures of different pore sizes and used these to study responses of a soil yeast to toxicant challenge.

## RESULTS

### Production of glass bead structures with different pore sizes.

**FIG 1:**
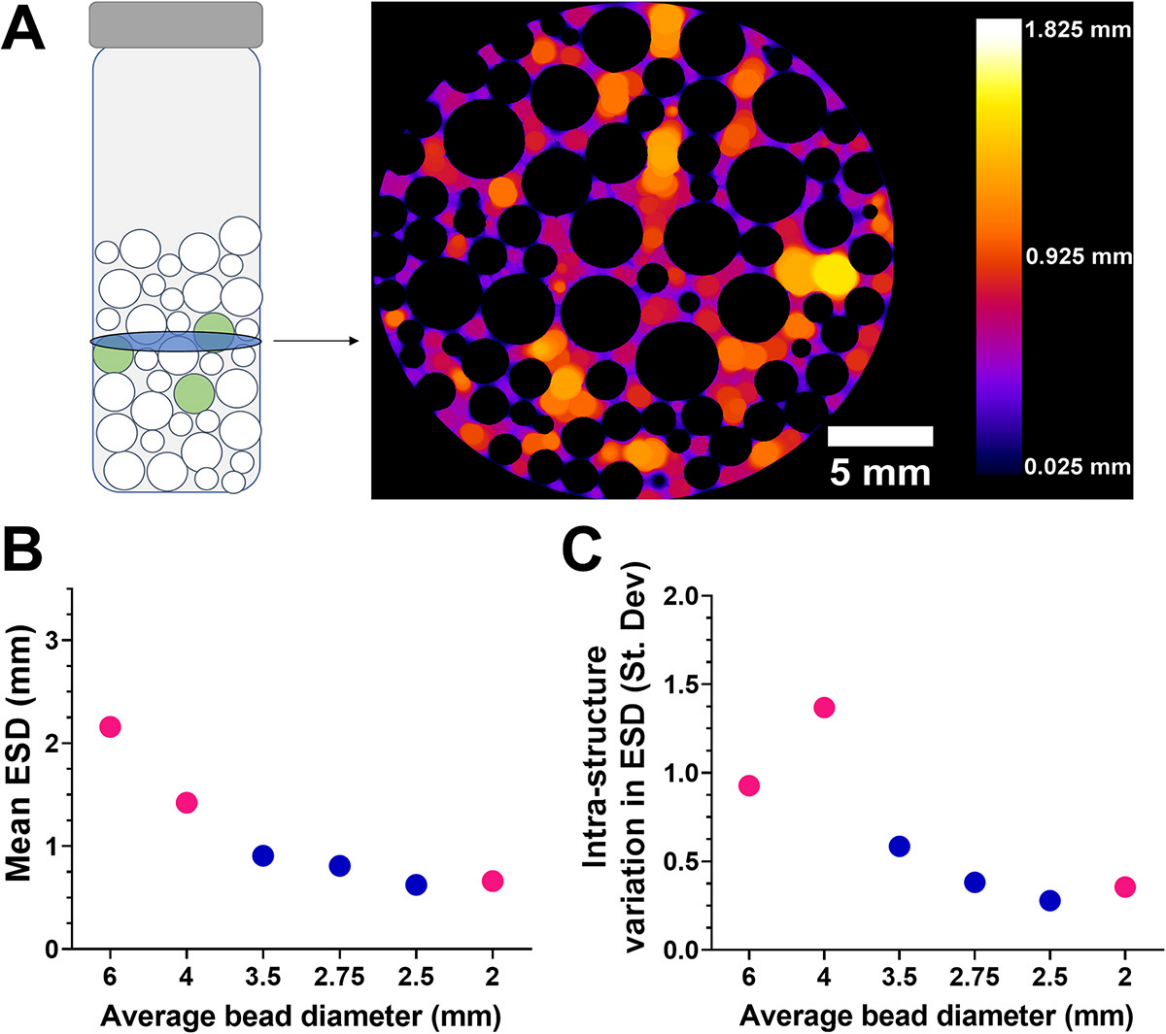
Production of structured environments of various pore size equivalent spherical diameters (ESDs). (A) Different sizes and ratios of glass beads were mixed in McCartney bottles to create structured environments (left). In the schematic, white circles indicate sterile glass beads, and green circles indicate beads surface inoculated with microbial cells (the yeast *S. podzolica*); bead/bottle dimensions are not to scale. Structures were X-ray CT scanned to determine pore space ESDs (right). Color scale represents the ESD in three dimensions, and black indicates solid (bead) volume. The example shown is a single X-ray slice of a structure comprising a mixture of 2-mm and 4-mm beads. (B) The pore space ESD across structures with various bead diameters. (C) The standard deviation of ESD within a structure, demonstrating that the level of such intrastructure ESD variation differs between the structure types. In panels B and C, pink circles represent monodisperse structures (i.e., structures composed of one particle size), and blue circles represent bidisperse structures of various proportions of 2- and 4-mm beads.

To investigate the impact of pore size on microbial resistance to an applied stress, we created structured environments by mixing different ratios of glass beads of different sizes. The total volume occupied by beads was adjusted to be constant across the different structures. The structures comprised either 6-mm, 4-mm, or 2-mm-diameter glass beads or different ratios of 4-mm and 2-mm beads (Fig. 1A). These different bead compositions proved sufficient to alter the average pore space equivalent spherical diameter (ESD) within the different structures. The ESD decreased with decreasing average bead diameter, determined by X-ray computed tomography (CT) (Fig. 1B and C). Furthermore, the variation in ESD among pores within a structure (intrastructure ESD) also tended to decrease with decreasing average bead diameter.

### Recovery of yeast from inoculated beads to assay stress resistance.

**FIG 2:**
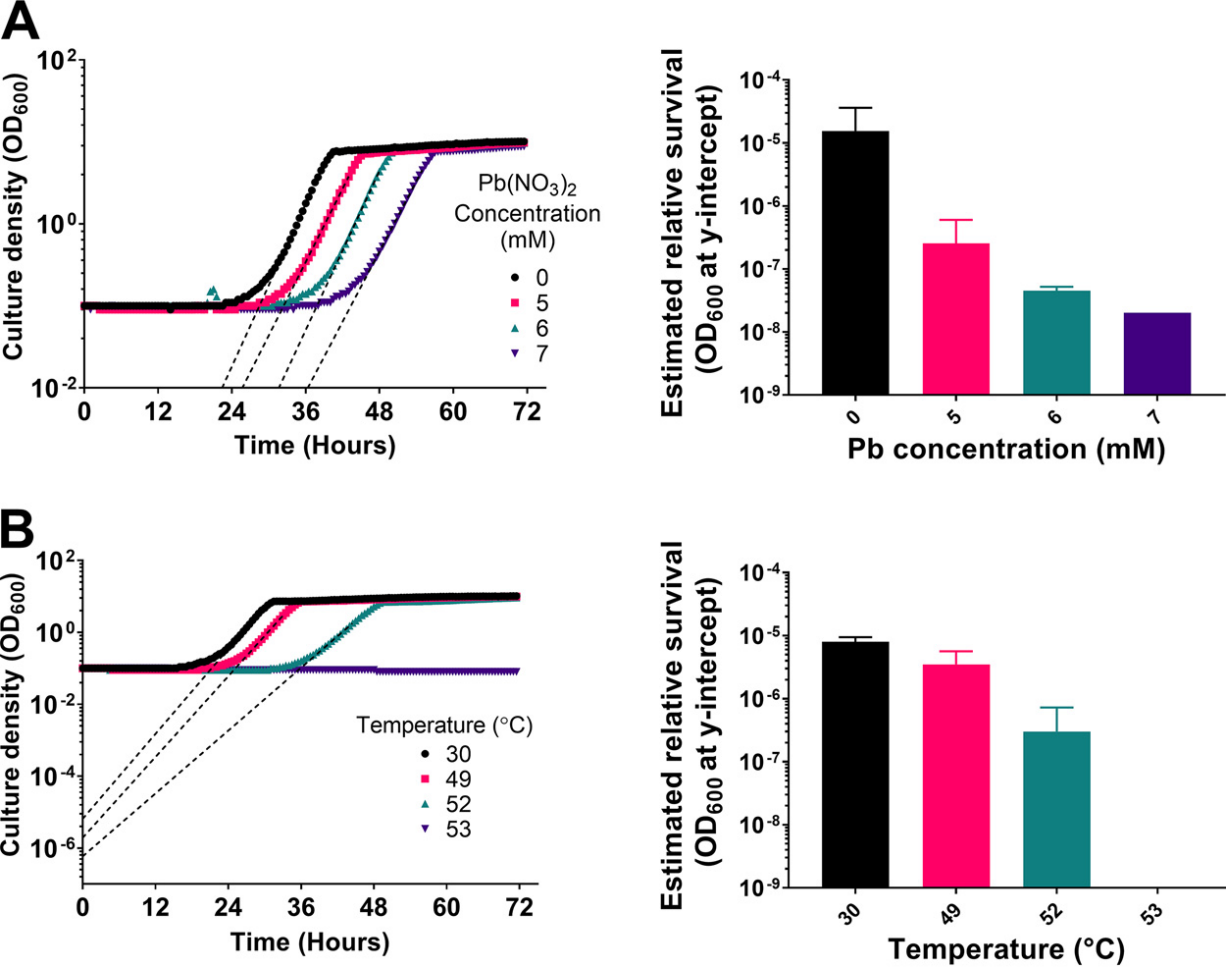
The *y*-intercept of cell outgrowth from beads negatively correlates with intensity of stress applied. Glass beads were coated with exponential-phase S. cerevisiae cells and exposed to a range of lead nitrate concentrations or temperatures. After 1 h, beads were washed and incubated in fresh medium for 5 h before transfer of supernatant (time zero) to 48-well plates and growth curves were generated. (A) Growth curves of bead outgrowth experiments after lead nitrate treatment (left) and corresponding *y*-intercepts derived from exponential regression of the exponential-phase growth slopes (right). (B) Growth curves of bead outgrowth experiments after heat shock treatment (left) and corresponding *y*-intercepts derived from exponential regression of the exponential-phase growth slopes (right). In growth curves, dashed lines are examples of the exponential regression used to determine *y*-intercept values. Growth curves are representative of those from two independent experiments; error bars in *y*-intercept graphs represent SEM of the two biological replicates.

Next, a method of introducing microorganisms into the glass bead structures was devised. This was achieved by coating sterile 5-mm glass beads with concanavalin A (ConA; a lectin carbohydrate-binding protein commonly used to adhere cells to glass substrates) by submerging them in a concanavalin A solution before drying them. These coated beads were subsequently inoculated with cells and incorporated within the structures (see Materials and Methods). First, to demonstrate that cells treated with a stressor could be recovered from coated glass beads, beads inoculated with the yeast model organism Saccharomyces cerevisiae were submersed in medium supplemented or not with lead nitrate. The beads were briefly washed and then transferred to fresh medium to allow surviving cells to grow into the medium, with back-extrapolation of resultant growth parameters serving to estimate that survival. The growth curves were analyzed by exponential regression to estimate the *y*-intercepts (after bead outgrowth) by extrapolation from the exponential phase of growth (22). These values were compared between treatments to estimate relative viable cell numbers in structures after the original treatments. Similar tests were also conducted with heat stress, as an alternative stressor that can be precisely controlled and rapidly stopped. The extrapolated *y*-intercept optical density at 600 nm (OD_600_) progressively decreased with increasing lead concentration and with increasing temperature (Fig. 2) (for presentation purposes, the extrapolation illustrated for lead stress is shown only down to *y* = 10^−2^ [Fig. 2A]). This observation of these anticipated relationships with the level of stress applied corroborated the use of the approach described with cell-inoculated glass beads for assaying relative survival. Next, this was scaled up to the bead structures, focusing on lead stress specifically.

### Environmental pore size impacts lead nitrate resistance.

**FIG 3:**
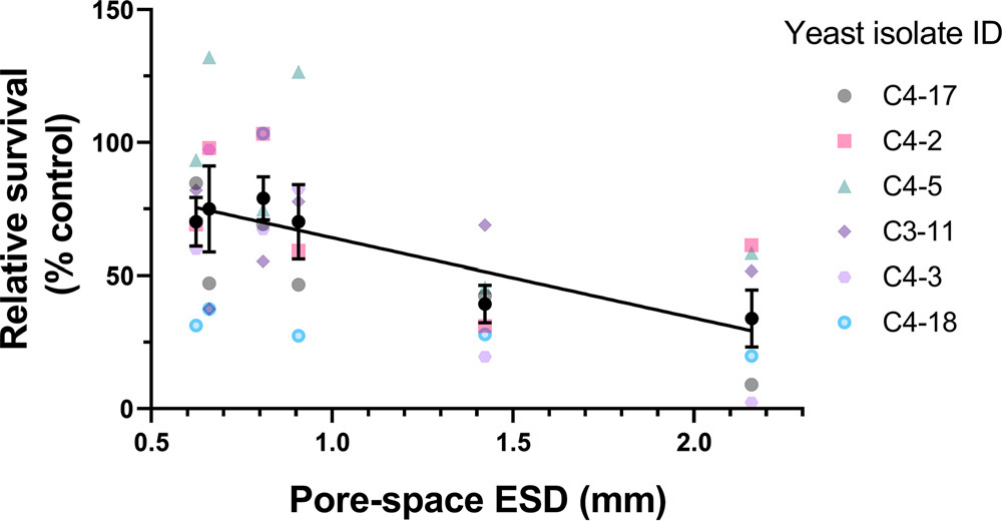
The survival of lead stress by S. podzolica in bead structures decreases with increasing pore size. Beads inoculated with exponential-phase cells of *S. podzolica* were introduced to a series of structures (three inoculated beads per structure) comprising glass beads of different sizes and various pore space diameters (Fig. 1) before challenge for 1 h with 9 mM lead nitrate in MYP medium including 30 mM succinic acid, pH 4.5. Cell survival was estimated from the *y*-intercepts of cell outgrowth from each structure poststress, calculated as a percentage of corresponding controls where water was added instead of lead nitrate. The individual colored points (means of three biological replicates) represent data for each of the six yeast isolates, with black points representing mean values across all the isolates ± SEM. The slope was fitted by linear regression (*R*^2^ = 0.277; *P = *0.001). ID, identifier.

Because the size of pores within a porous structure can alter the rate of movement and diffusion of fluids, we hypothesized that microorganisms within structures of different pore sizes would be differentially exposed to chemical stressors, reflected in differences in survival. To compare stress resistance of cells in structured glass bead environments, stress survival experiments were conducted using inoculated beads introduced to six types of structure and across six different wild isolates of the soil yeast Saitozyma podzolica. Cells were exposed in each structure to MYP medium (7% malt extract [Sigma], 0.5% yeast extract [Oxoid], 2.5% Soytone [BD Bacto]) supplemented with succinic acid and either 9 mM lead nitrate (treatment) or an equivalent volume of water (control). A higher concentration of lead nitrate was used in this study, as S. podzolica demonstrated a higher lead resistance than S. cerevisiae used as described above. Simple linear regression indicated that there was a significant negative relationship between pore ESD and posttreatment survival across the six yeast isolates (each in biological triplicate) (*R*^2^ = 0.277; *P < *0.001). That is, the survival of *S. podzolica* under lead stress was decreased with increasing pore space ESD (Fig. 3).

**FIG 4:**
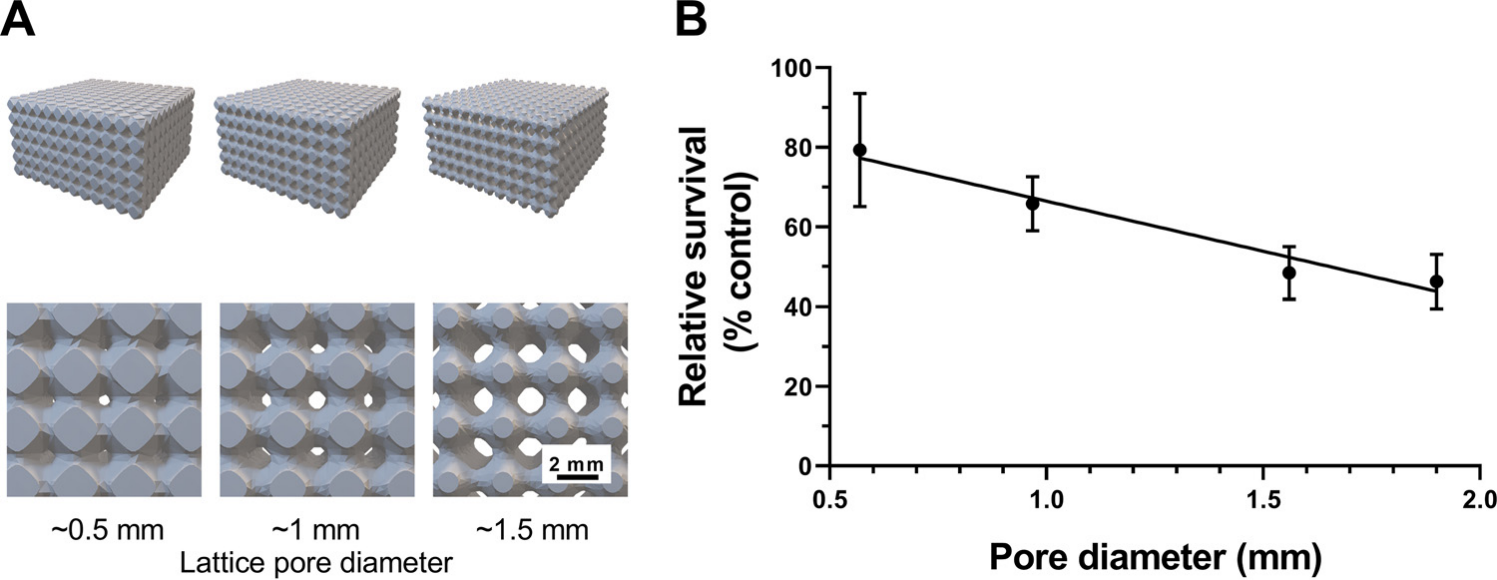
The survival of lead stress by *S. podzolica* in lattice structures decreases with increasing pore diameter. Additively manufactured lattice structures with four different pore diameters (0.5 mm, 1 mm, 1.5 mm, and 1.9 mm) (computer models shown in panel A) were inoculated with exponential-phase *S. podzolica* cells (isolate C4-17), which were subsequently challenged with 9 mM lead nitrate in MYP medium including 30 mM succinic acid, pH 4.5. (B) The percent survival (OD_600_ *y*-intercept) of *S. podzolica* in each structure was determined against corresponding controls where water was added instead of lead nitrate. Mean results are shown from three biological replicates; error bars represent SEM. The slope was fitted by linear regression (*R*^2^ = 0.485; *P* = 0.008).

We noted that there was a general correlation between mean pore size and the extent of variation in pore sizes within the glass bead structures (Fig. 1B and C). To distinguish between the potential contributions of these two variables to effects on microbial survival, lattice structures were produced by additive manufacturing. Each lattice (dimensions, 60 mm wide by 60 mm deep by 30 mm high) was composed of repeating units of the same size, producing a consistent pore diameter across each structure. The sizes of repeating units were designed to be different for different lattices manufactured, giving different pore diameters between lattices but uniform within any lattice, unlike the glass bead structures. These structures were inoculated by submersion in *S. podzolica* broth culture before replacement of the medium with lead nitrate-supplemented broth. Here, a single representative isolate (C4-17) was used to inoculate lattices. Similar to the results from the bead experiments, simple linear regression indicated that there was a significant negative relationship between lattice pore diameter and *S. podzolica* survival posttreatment (*R*^2^ = 0.485; *P = *0.008) (Fig. 4). The use of the lattice structures ruled out the possibility that this result was related to intrastructure pore size variation.

Because each lattice had the same overall dimensions and the same total number of pores but differed in pore diameter, the proportion of lattice occupied by pore space increases with increasing pore diameter in the different designs. Given also that the starting inoculum per lattice (i.e., numbers of cells retained on lattice surfaces after washing out the broth inoculum) was similar across lattices—as indicated by similar levels of outgrowth in control tests with lattices of different designs (data not shown)—it follows that the number of yeast cells per unit volume of pore space was decreased with increasing pore diameter in the different lattices. Accordingly, cell densities were calculated as 95, 12, and 3.2 cells per μl of pore space for lattices with pore diameters of 500, 1,000, and 1,500 μm, respectively.

Because of these differences in cell density per unit pore space in the different structures, we considered the possibility that differential depletion of lead (arising from lead uptake by the cells) could have given rise to differences in final lead concentrations and hence in the relative levels of stress exerted in the different structures. However, we found that lead uptake at cell densities reproducing those attained in the structures (described above) were not sufficient to cause any significant lead depletion from the medium (see Fig. S1 in the supplemental material).

**FIG 5:**
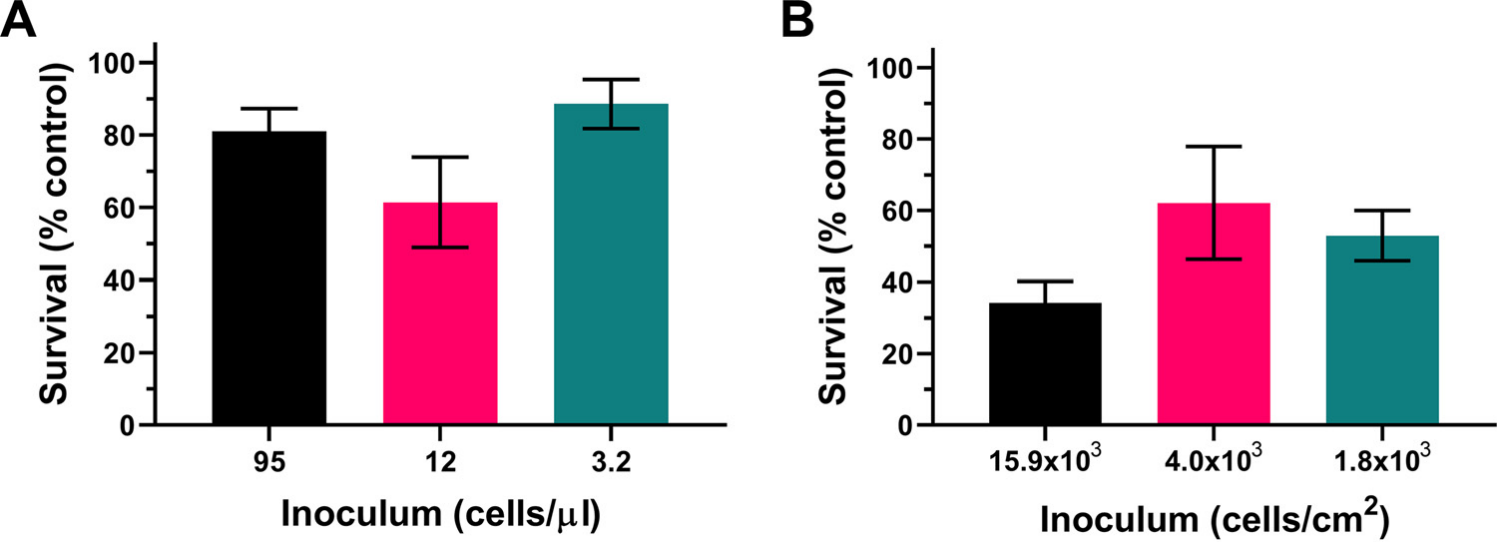
Assessment of the potential impact of cell density on lead resistance. The survival rates of *S. podzolica* in response to a 9 mM lead nitrate challenge for 1 h at cell densities equivalent to those calculated for either pore volume (A) or pore surface area (B) of lattice structures with pore diameters of 500 μm (black), 1,000 μm (pink) and 1,500 μm (teal) are shown.

To test further for a possible contribution of cell density effects to the present observations, we examined directly whether the different attained densities could influence survival of lead stress. The estimated cell densities in the different lattice designs (described above) were again reproduced by appropriate dilution of broth cultures (in 15-ml Falcon tubes), omitting the lattice structures. The results showed no significant difference in percent survival of lead at these different cell densities (*P = *0.166, one-way analysis of variance [ANOVA] with Tukey’s multiple-comparison test) (Fig. 5A). Figure 5A suggests possible lower survival at the intermediate cell density, but no trend similar to that seen in the structured-environment experiments was evident. Finally, since many or most cells would be adhered to (lattice pore) surfaces in the structured environments, we assayed specifically the possibility of an effect of surface density of cells on lead resistance. An experiment similar to that described above was conducted, but in this case with cells adhered to the bottoms of microplates (see Materials and Methods) and at densities calculated by considering pore surface area in the lattice structures rather than volume (this calculation gave densities of ∼15.9 × 10^3^, 4.0 × 10^3^, and 1.8 × 10^3^ cells/cm^2^ for the lattices with pore diameters of 500, 1,000, and 1,500 μm, respectively). Similar to the observations with cells in suspension, there was no significant effect of surface-adhered cell density on lead nitrate resistance (*P = *0.243) (Fig. 5B). (A hint of possible lower survival at the highest cell density, corresponding to the smallest pore size in lattices, did not reflect the trend in the lattice experiments [Fig. 4].) The results collectively indicate that cell density effects do not contribute to the observations from the structured environment experiments, presented in Fig. 3 and 4. This reinforces the inference that it is differences in pore size, specifically, which determined yeast survival of the toxic metal stress.

## DISCUSSION

In this study, we examined the stress resistance of a natural soil yeast incorporated into laboratory-constructed porous environments of various pore diameters. The results showed that increases in the average equivalent spherical diameters (ESDs) of a structure’s porous architecture are associated with decreased survival of cells to toxic metal challenge. This was evident in both glass bead and additively manufactured lattice model structures, methodologies developed as part of this work. The findings enabled by exploitation of these new tools highlight the importance of the physical architecture of an organism’s immediate environment for its response to environmental perturbation.

Glass beads of different diameters (and mixed at different ratios) were used to produce structures with different mean pore ESDs, a measure of pore size. Furthermore, we demonstrated that individual glass beads could be inoculated with yeast, exposed to a stressor (lead nitrate or heat in this study), and then subsequently recovered and cells grown out to determine relative survival. Glass beads inoculated with bacteria have previously been used to study denitrification in porous environments (7), and beads have also been used as a solid interface to cultivate bacteria and study differentiation on solid surfaces (23). This report presents a simple method for making and incorporating organisms into three-dimensional (3D) structures of glass beads, enabling investigation of interrelationships between microbial activity and environmental structure. In addition, we demonstrated that additively manufactured lattices with uniform pore diameter could also support inoculation and subsequent recovery of yeast cells. This recent, accessible manufacturing approach gave precise control of technical reproducibility between structures across experiments while offering uniform structures with a single pore size (although these pore sizes could be varied within a lattice if desired).

Across the structures produced in this study with either method, pore diameters ranged from ∼0.5 mm to ∼2.1 mm and so are within the range found typically in soils (24, 25), building materials (26, 27), and biomedical materials such as tissue scaffolding (28). If desirable, it should be possible to produce structures with smaller (or larger) pore sizes by using smaller beads or higher-resolution 3D printing technology. Therefore, whereas we employed these structures to ask questions relevant to microorganisms in soil habitats, i.e., inoculation with a common soil yeast and exposure to soil-relevant stressors (e.g., metal pollutant and temperature), the methodology is adaptable for investigating other types of porous environments.

The glass bead and lattice structures were used to test the hypothesis that increased porosity of structures with larger pores may result in increased exposure (hence decreased survival) of cells to a chemical stressor compared to that of cells within smaller pores. Results from both types of structures substantiated this hypothesis, determined using yeast inocula exposed to a defined concentration of lead nitrate. Whereas we noted that the distribution of pore ESDs within bead structures tended to broaden with increasing mean ESD, the survival trends in the bead experiments were reproduced in the lattice experiments where there was a uniform pore size within each lattice. This indicated that the survival trend was related to increasing mean pore size specifically.

It was noted that cell densities within pores of different sizes differed in our structure designs. Cell density is reported to influence stress resistance in other systems (29–31). However, the present survival difference between structures could not be explained by cell density effects, as replication of the different cell densities in separate assays showed no significant differences in survival (or in stressor depletion from the medium). This corroborated that it is specifically the physical pore size of structures that impacted stress resilience here. Given that, it could be of interest to test the influence of other pore parameters in a similar way in the future, such as pore shape or pore connectivity (32).

As there is no fluid flow (either laminar or turbulent) in the structured environments used in this study, lead depleted from a cell’s immediate environment by cellular uptake is predominantly replaced by diffusion (33), and diffusivity in porous media is dependent on porosity (the proportion of the environment occupied by pore space) (34). As the present structures retained the same total volume over different average ESDs (or pore diameters), porosity within these structures will increase as pore ESD increases. Therefore, we infer that lead depleted by local cellular uptake is subsequently replenished at a rate related to structure porosity. As a consequence, greater diffusivity in structures with larger pores would elevate the mean (over time) stressor exposure of a cell. The present results support this model. It could be interesting to investigate this model further in the future, for example, with direct assay of dynamic stressor exposure of cells. As a proxy, in this study, we attempted to compare lead accumulation by cells within the different lattice structures. However, the lattice material itself adsorbed significant amounts of lead (data not shown), confounding the potential for distinguishing lead uptake specifically by cells within the lattices.

### Conclusions.

The physical environments in which microorganisms naturally reside are rarely unstructured, but the impacts of environmental structure are not usually captured in laboratory experiments (35). Soil structure in particular is a dynamic characteristic that is variable between different soil types, locations, and management practices in agricultural soils (36, 37), with pore size being a key parameter. The results of the present study show that changes in the pore size of a microorganism’s (micro)environment influence its resistance to stress, demonstrated here with lead as a soil-polluting metal stressor (which arises from mining and smelting activities [38]). Hence, when assessing the impact of environmental stressors on microorganisms, the environmental architecture in which such organisms naturally reside should be considered and even incorporated into experimental design where appropriate, as evidently physical structure alone can impact microbial stress resistance. Such endeavors are enabled by new tools for introducing environmental structure to laboratory studies, such as those utilized and developed in this investigation.

## MATERIALS AND METHODS

### Yeasts and culture conditions.

The haploid Saccharomyces cerevisiae strain BY4741 (*MAT***a** *his3-1 leu2-0 met15-0 ura3-0*) was maintained and grown in YPD medium (2% peptone [Oxoid], 1% yeast extract [Oxoid], 2% d-glucose). Isolates of *Saitozyma podzolica*, identified by internal transcribed spacer (ITS) sequencing and randomly amplified polymorphic DNA (RAPD) PCR as described by Holland et al. (39), were recovered from soil near a disused metal smelting works in the northeast of the United Kingdom (http://www.twsitelines.info/SMR/4192). *S. podzolica* was maintained and grown in MYP medium (7% malt extract [Sigma], 0.5% yeast extract [Oxoid], 2.5% Soytone [BD Bacto]). Where required, media were solidified with 2% (wt/vol) agar. For experiments, single colonies were used to inoculate 10 ml of medium in 50-ml Erlenmeyer flasks and incubated with orbital shaking (New Brunswick Scientific) at 120 revolutions min ^−1^, either at 30°C for S. cerevisiae or at 24°C for *S. podzolica*.

### Inoculation of glass beads with yeasts.

Autoclaved glass beads measuring 5 mm in diameter (Dixon Science) were submersed in 2 mg/ml of concanavalin A (ConA; Sigma) dissolved in sterile deionized water and were subsequently dried at room temperature. Following this, the ConA-coated beads were submersed for 10 min in a culture (OD_600_ ∼ 1.0) of exponential-phase yeast cells, with gentle agitation every 2 min. For experiments, beads were briefly rinsed in fresh medium to remove excess culture medium and nonattached cells before use of the beads.

### Preparation of glass bead structures.

Glass beads of different sizes (Sigma) were used. For monodisperse structures (structures composed of one particle size), McCartney bottles were filled with either 2-, 4-, or 6-mm-diameter beads up to a volume of 5 cm^3^. For bidisperse structures, beads of 2- and 4-mm diameters were mixed to give either 1:3, 1:1, or 3:1 ratios by volume of the two bead types, to a total volume of 5 cm^3^. After autoclaving, bottles of either monodisperse or bidisperse structures were shaken and three 5-mm beads inoculated with cells (described above) were placed on the sterile glass beads within the bottle, before a further 5-cm^3^ volume of the same type of bead or bead mix was decanted on top from a separate bottle. This produced an ∼10-cm^3^ structure, with cell-inoculated beads positioned in the middle of the structure and an even mix of beads above and below these. This method prevented the cells on the inoculated beads from being dislodged by the vigorous shaking using to mix the two batches of mixed beads before they were combined.

### X-ray CT and image analysis.

The pore structures of glass bead structures were imaged using a Phoenix Nanotom S X-ray computed tomography (CT) scanner at the Hounsfield Facility, University of Nottingham. Voxel resolution was set at 12.5 μm, potential energy at 90 kV, and current at 75 μA. The total scan time was 15 min per structure, and a total of 1,200 projections were captured for each structure. VGStudio MAX was used to generate 3D volumes of CT images, crop images, and export images as stacks. Images were exported as image stacks to ImageJ-Win64, where they were binarized using the Li threshold algorithm without any prior filtering due to the high quality of the original images. Total porosity and equivalent spherical diameter (ESD) were determined using the BoneJ plugin (40).

### Stress resistance assays.

To determine whether inoculated beads could be used in stress and cell recovery assays, 5-mm beads inoculated with S. cerevisiae cells were transferred to 2-ml Eppendorf tubes with submersion in 1.5 ml of yeast extract-peptone-dextrose (YEPD) culture medium either supplemented with different lead nitrate concentrations and incubated for 1 h at 24°C or heat stressed at different temperatures for 10 min. Beads were rinsed in fresh medium before assessment of cell survival (see below).

To assess the impact of the different glass bead structures on stress survival, 10 ml of MYP medium supplemented with 30 mM succinic acid (final pH 4.7) and 9 mM lead nitrate (the latter being omitted in controls) was added to structures prepared as described above. After 1 h, the cell-coated beads were recovered by decanting the medium, pouring beads onto a flat, sterile surface, and picking out the 5-mm beads with sterile tweezers. The three beads from each structure were briefly rinsed in fresh MYP medium to remove residual lead before assessing cell survival.

### Outgrowth of organisms from glass beads.

To assess the relative survival of cells, glass beads recovered from stress treatment experiments were transferred to wells of a 24-well microtiter plate (Greiner Bio-One), with each well containing 350 μl of fresh medium and one added bead. The plates were incubated for 5 h at 24°C and 120 revolutions min^−1^ to allow bead-adhered cells to divide into the medium. An aliquot (300 μl) of the medium, now containing cells, was transferred to a 48-well microtiter plate, and further growth was monitored during incubation at 24°C with shaking in a BioTek Powerwave XS microplate spectrophotometer and OD_600_ measurement every 30 min. Resulting growth curves were used to infer back to a theoretical starting OD using multiple regression (22).

### Additive manufacturing of lattice structures.

FLatt Pack V 1.3 was used to produce lattice structures with pore diameters similar to those of bead structures (41). Lattices were made using the “cuboid” geometry setting, and the “primitive” cell type (under the “network” phase selection) was used as the repeating cell of the lattice. Each lattice was composed of 12 repeating cells in the Y plane and 6 repeating cells in the X and Z planes. The overall lattice dimensions were set to 30-mm width by 30-mm length by 15-mm height. Different pore diameters were produced by altering the width (thickness) of the lattice walls, such that increasing the wall thickness reduced pore size. Lattices were printed at the Advanced Manufacturing Facility, University of Nottingham, on a Formlabs Form 2 SLA 3D printer, using Formlabs clear resin (product code RS-F2-GPCL-04). This resin was chosen because it allows printing at a resolution of 25 μm.

### Stress survival assays in manufactured lattices.

Lattices were sterilized with isopropanol (70% [vol/vol]) and then coated with ConA (2 mg/ml) and dried at room temperature. ConA-coated lattices were submersed for 10 min in a culture (OD_600_ ∼ 1.0) of exponential-phase yeast cells, with agitation every 2 min, before being washed in MYP medium to remove nonadherent cells. Subsequently, lattices were submersed in MYP medium supplemented with 30 mM succinic acid (to a final pH of 4.7) and 9 mM lead nitrate (the latter being omitted in controls). After 1 h, lattices were washed with MYP medium, transferred to 40 ml of fresh MYP medium, and then incubated for 5 h at 24°C with shaking at 120 revolutions min^−1^. Subsequently, 300-μl volumes of supernatant samples were transferred to wells of a 48-well microtiter plate and outgrowth was measured as in the bead outgrowth experiments described above.

### Assay for effects of cell density.

To test for any effect of different cell densities on lead depletion from the medium, the cell density in each lattice structure was calculated as the estimated number of cells per volume of pore space, by first calculating the estimated number of cells per lattice from outgrowth OD_600_ and then dividing this number by the known volume of pore space. Then, 15-ml Falcon tubes containing 10 ml of MYP medium supplemented with 30 mM succinic acid and 9 mM lead nitrate (or without lead nitrate in controls) were inoculated to the different, calculated cell densities with exponential-phase *S. podzolica* cells. After incubation for 1 h, cell suspensions were syringe filtered through a 0.2-μm filter to separate cells and colloidal material from the medium. Filtered medium was subsequently diluted using 2% (vol/vol) nitric acid to reach the appropriate analytical range for multielement analysis by inductively coupled plasma mass spectrometry (ICP-MS). ICP-MS was conducted using a Thermo Fisher Scientific iCAP-Q equipped with collision cell technology with energy discrimination (CCTED) and analyzed using Qtegra software (Thermo Fisher Scientific).

To examine the effect of cell suspension density on survival of lead exposure, cell densities and stress conditions were used as described above, but with cell suspensions prepared in 500-μl volumes in wells of a 48-well microplate. After 1 h of lead exposure, cell suspensions were diluted to decrease Pb to subinhibitory concentrations and 300 μl of diluted cell suspension was transferred to wells of a 48-well plate to monitor outgrowth and determine *y*-intercepts from resulting growth curves, as outlined above.

To examine the effect of surface-bound cell density on lead survival, the number of cells per lattice was calculated as before and divided by total pore surface area to estimate the number of cells per unit surface area. Then, wells of a 48-well plate coated with dried ConA (2 mg/ml) were inoculated with 500 μl of cell suspension containing a total number of cells sufficient to coat the bottom of the well at the desired cell/surface area (∼15.9 × 10^3^, 4.0 × 10^3^, and 1.8 × 10^3^ cells/cm^2^ for the lattices with pore diameters of 500, 1,000, and 1,500 μm, respectively). Plates were then centrifuged at 3,000 × *g* for 3 min to force cells to the bottom of the well before aspirating the medium and replacing it with the same volume of MYP medium supplemented with 30 mM succinic acid and 9 mM lead nitrate (or without lead nitrate in controls) and incubating the plates for 1 h. Posttreatment, plates were centrifuged as before and wells were washed twice with sterile water to remove residual lead, before adding 350 μl of fresh MYP medium and growing cells for 5 h. Then, 300-μl samples of culture outgrowth were transferred to a new 48-well plate for continued outgrowth and *y*-intercept analysis as described above.

### Statistical analysis.

Linear regression was performed with GraphPad Prism version 8.2.1, accounting for replicates and sample size, with a significance threshold at a *P* value of 0.05. Comparisons between treatments for lead concentration assays and cell density experiments were analyzed using an unpaired *t* test or one-way ANOVA for multiple comparisons, with a significance threshold at a *P *value of 0.05.
