# Supplementary material for: Microbial Metal Resistance within Structured Environments Is Inversely Related to Environmental Pore Size
Source: Appl Environ Microbiol. 2021 Sep 28;87(20):e01005-21. doi: 10.1128/AEM.01005-21 (PMC8478441; doi:10.1128/AEM.01005-21)
Supplement: Supplemental file 1 — Figure S1. Download AEM.01005-21-s0001.pdf, PDF file, 0.06 MB [file aem.01005-21-s0001.pdf]

SUPPLEMENTAL MATERIAL

Microbial Metal Resistance within Structured Environments is Inversely Related to Pore Size

HJ Harvey et al.

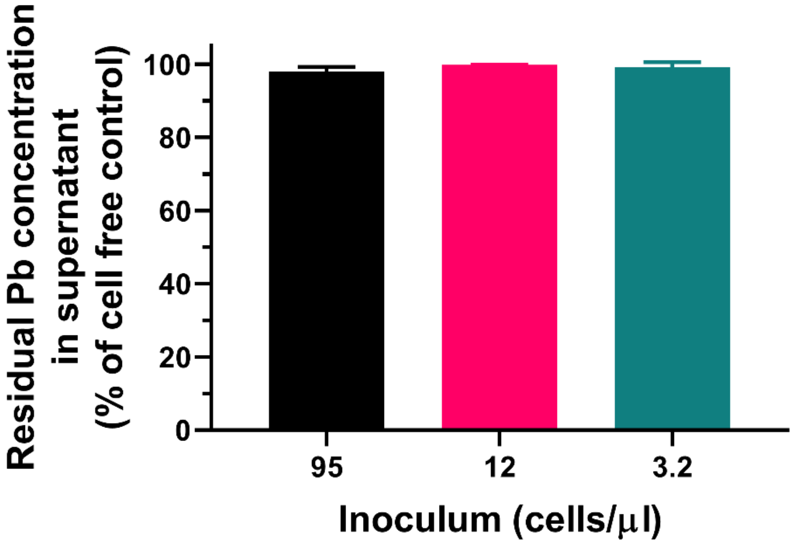

Supplementary Figure 1- Analysis of lead recovered from supernatant containing different cell densities.

Cells of *S. podzolica* in 24-well plates were exposed to 9mM lead nitrate and the lead level in the supernatant determined after 1 hr incubation. with The cells were supplied at cell densities equivalent to those calculated for lattice structures with pore diameters of 500 µm (black), 1000 µm (pink) and 1500 µm (teal). Pb concentrations after 1 hour are expressed as percentages of no-cell controls.
